# Delivery of SA35 and SA40 peptides in mice enhances humoral and cellular immune responses and confers protection against *Cryptosporidium parvum* infection

**DOI:** 10.1186/s13071-019-3486-8

**Published:** 2019-05-15

**Authors:** Fabio Tosini, Alessandra Ludovisi, Daniele Tonanzi, Marco Amati, Simona Cherchi, Edoardo Pozio, Maria Angeles Gómez-Morales

**Affiliations:** European Union Reference Laboratory for Parasites, Istituto Superiore di Sanità, Rome, Italy

**Keywords:** *Cryptosporidium*, Cpa135, gp900, Mucosal immunisation, Maternal transfer, Recombinant antigen

## Abstract

**Background:**

*Cryptosporidium parvum* is a major cause of diarrhea in children and ruminants at the earliest stages of life. Maternal antibodies represent the main shield of neonate mammals for most of the infections. Two recombinant antigens (SA35 and SA40), portions of two *C. parvum* proteins, were tested for their ability to induce immune responses in adult mice and for protection on neonate BALB/c mice born from females immunised by mucosal delivery of both peptides.

**Methods:**

Adult BALB/c mice were intraperitoneally immunised with SA35 and SA40, separately or mixed, and their immune response was characterised. Furthermore, BALB/c pregnant mice were immunised by mucosal delivery with an SA35/40 mix, before and during pregnancy. Soon after birth, their offspring were infected with two doses (1 × 10^5^ and 5 × 10^3^) of *C. parvum* oocysts and the parasitic burden was determined at 5 and 9 days post-infection.

**Results:**

Intraperitoneal immunisation with SA35 and SA40 induced specific IgG and IgG1 in serum, specific IgA in the intestinal mucosa, increase of CD3+/CD4+ and CD30+ cells in splenocytes, which produced IFN-γ. Neonates born from immunised mice and infected with 1 × 10^5^ oocysts showed a significant reduction of oocysts and intestinal forms (23 and 42%, respectively). A reduction of all parasitic forms (96%; *P* < 0.05) was observed when neonates were infected with 5 × 10^3^ oocysts.

**Conclusions:**

SA35 and SA40 peptides induce specific humoral and cell-mediated immune responses to *C. parvum* in adult mice. Moreover, mucosal administration of the SA35/40 mix in pregnant mice reduces *C. parvum* burden in their litters.

## Background

The *Cryptosporidium* genus includes 38 species that infect a wide range of vertebrates, including many mammals. Humans are also susceptible to these parasites, and domesticated and wild ruminants, particularly livestock, represent important sources of zoonotic transmission [1]. In mammals, *Cryptosporidium* spp. mainly affect the gut, causing gastrointestinal symptoms and diarrhoea. Approximately 90% of human infections are caused by *Cryptosporidium parvum* and *Cryptosporidium hominis*, although over 20 species can infect humans [2]. In immunocompetent patients, cryptosporidiosis is a self-limiting infection of the small intestine that causes watery diarrhoea and can last up to ten days, whereas the infection can become a chronic and life-threatening disease in immunocompromised individuals such as AIDS subjects [3].

Transmission occurs *via* the faecal-oral route through the ingestion of oocysts contaminating food or water. Oocysts are highly resistant to environmental conditions, chlorination and other sterilisation treatments, and contamination of water plants can therefore cause massive outbreaks [4]. It follows that the Food and Agriculture Organisation and the World Health Organisation consider protozoa of the genus *Cryptosporidium* to be one of the most significant food-borne parasites [5].

In livestock husbandry, *C. parvum* is a major cause of severe diarrhoea among neonate calves and lambs, resulting in substantial costs for farmers. A recent study on diarrhoea conducted on calves younger than one month reported that *C. parvum* is responsible, as the sole agent, for 37% of diarrhoeal infections and for 20% of co-infections with other intestinal pathogens [6].

In recent years, infections by *C. parvum* and *C. hominis* have emerged as significant causes of infantile diarrhoea. An extended case-control study, referred to as the Global Enteric Multicenter Study (GEMS) [7], has shown a high prevalence of *Cryptosporidium* spp. among children in developing countries, ranking these protozoa among the four pathogens responsible for the majority of diarrhoea cases in children younger than five years [8]. The prevalence of these parasites is higher in infants aged less than 11 months and, in this age range, *Cryptosporidium* spp. is the second most common cause of death associated with diarrhoea, after rotavirus [9].

The susceptibility of neonates and children to *Cryptosporidium* spp. is not completely understood. In the nursing period, the innate immune response of enterocytes, which is usually triggered by the stimulation of toll-like receptor 4 (TLR4), is almost completely inhibited so as to favour the establishment of commensal flora in the gut [10]. Given that the immune response to *Cryptosporidium* spp. begins with TLR4 stimulation and the consequent activation of the NF-kappa B pathway [11], the temporary inhibition of the innate enterocyte response could promote the proliferation and dissemination of *Cryptosporidium* spp. in the neonatal intestine.

At present, nitazoxanide is the only drug approved for cryptosporidiosis by the US Food and Drug Administration (US-FDA), but this drug cannot be used in children younger than one year and is ineffective in immunodeficient patients [12].

A vaccine for cryptosporidiosis is not yet available, and immune protection of neonates is difficult to achieve because of their early-age immune status. However, various *Cryptosporidium* spp. proteins, particularly those considered to be virulence factors, have been proposed as possible vaccine candidates [13, 14].

In this context, the passive immunisation of neonates might be an alternative to a conventional vaccine. Indeed, passive immunity provided through hyperimmune bovine colostrum has been shown to establish an appreciable level of protection in human patients with AIDS-associated cryptosporidiosis [15, 16]. More recently, the protective role of maternal anti-*Cryptosporidium* antibodies has been demonstrated in two natural contexts. A survey of a child cohort in Bangladesh reported that the presence of anti-*Cryptosporidium* IgA in breast milk protects neonates from cryptosporidiosis [17]. A second study, conducted in an endemic area of Tanzania on a cohort of breastfeeding mothers and their infants (0 to 6 months), has shown that the infection rate increases with a reduction in maternal milk ingestion [18].

This study investigates the feasibility of an immunological preventive treatment to impede or reduce cryptosporidiosis in newborn mammals. As a preliminary step, two recombinant antigens, SA35 and SA40 [19], were inoculated in adult mice to achieve the onset of a specific immune response. A group of female mice were then immunised by mucosal delivery of the same antigens prior to and during their pregnancies in parallel with a control group treated only with the adjuvant. The offspring of these mice were then infected with two different doses of *C. parvum* oocysts (1 × 10^5^ and 5 × 10^3^) and the parasite burden was measured by flow cytometry, microscopic count and a quantitative PCR assay. Our results indicated that neonates born from immunised mothers had a reduced parasitic load compared to the neonates born from the control mice.

## Methods

### Parasites and reagents

*Cryptosporidium parvum* oocysts (isolate code ISSC6) were collected from faeces of experimentally infected calves and purified by centrifuging with sucrose and Percoll^®^ (Sigma-Aldrich, Saint Louis, MO, USA) density-gradients [20]. *Cryptosporidium parvum*-crude extract (CCE) was obtained as previously described [21]. Heat-labile enterotoxin (LT) was obtained from Chiron SpA (Siena, Italy), and ketavet and xylazine solutions were provided by the Animal Care Unit of the Istituto Superiore di Sanità in Rome, Italy. A rabbit anti-*C. parvum* IgG polyclonal antibody, which recognises all stages of *C. parvum* [22], was used for IFA assays.

### Recombinant antigens

The recombinant peptides SA35 (MW, 35 kDa) and SA40 (MW, 40 kDa) represent antigenic portions of two microneme proteins of *C. parvum* named Cpa135 (cgd7_1730) and Gp900 (cgd7_4020), respectively [23, 24]. Protein ID codes refer to sequences in the CryptoDB database, release 37 [25]. The two peptides were purified as previously described [26], dialysed in phosphate buffered saline (PBS) plus 50% glycerol and stored in aliquots at −20 °C. Both peptides were used as a sole immunising agent or as a 1:1 mixture of the two antigens (referred to hereafter as the SA35/40 mix).

### Experimental model

Eight- to ten-week-old BALB/c female mice (Charles River Laboratories, Milan, Italy) were housed in the Animal Care Unit of the Istituto Superiore di Sanità in accordance with European Directive 63/2010. The *in vivo* protocol was approved by the Italian Ministry of Health (approval no. 8/2014-B, 15 January 2014).

### Immunisation protocol for antigen characterisation

Three groups of five female mice each were immunised intraperitoneally: one group with 5 μg of SA35, a second group with 5 μg of SA40, and a third group with the SA35/40 mix (5 μg plus 5 μg of SA35 and SA40, respectively). A control group (*n* = 5) was injected with an equal volume of PBS instead of antigens, according to the same immunisation schedule, which was as follows: day 0, peptides or PBS were administered in Freund’s complete adjuvant (Sigma-Aldrich); day 14, the same amount of peptides or PBS were inoculated using Freund’s incomplete adjuvant (Sigma-Aldrich); day 28, the last booster was provided using the same quantity of peptides or PBS without adjuvant. The mice were bled on days 0, 7, 14, 21, 28 and 35 following initial immunisation. Individual sera were stored at −20 °C. Faecal samples for an IgA assay were collected weekly from each mouse separately, weighed and dissolved in 1.0 ml of PBS with 0.1% sodium azide per 100 mg of faecal material, and a cocktail of protease inhibitors (Sigma-Aldrich) was added at a ratio of 1:20 to each sample. The samples were then vortexed for 5–10 min and centrifuged to remove debris; supernatants were collected and stored at −70 °C.

### Detection of *C. parvum* antibodies in serum samples and faecal pellets

Levels of IgG, IgG1 and IgA in serum samples and IgA in faecal samples specific to *C. parvum* were determined by ELISA. Levels of IgG and IgA specific to SA35 and SA40 peptides were also determined by ELISA. Microtiter plates (Nunc-Immuno Plate Polysorp™, Sigma-Aldrich, Roskilde, Denmark) were blocked using 200 μl per well of 1% w/v BSA in PBS, at room temperature (RT) for 90 min and coated with 1 µg/ml of recombinant peptides (SA35 or SA40) or 5 µg/ml of CCE in a 50 mM carbonate/bicarbonate buffer with a pH of 9.6, and incubated at 37 °C for 90 min. Serum samples were diluted in PBS with 0.5% w/v BSA and 0.05% v/v Tween-20. Supernatants of faecal samples were diluted as described above. Plates were washed three times with 0.05%-Tween-20 in PBS after incubation with sera, and washes were repeated after incubation using conjugated antibodies. Dilutions of 1:50 for sera and 1:10 for faecal supernatants were established to be the optimal working conditions. These dilutions were then added in duplicate to wells and incubated overnight at 4 °C. As negative controls, the corresponding pre-immune faecal and serum samples from each mouse were used at the same dilution in ELISA. Bound antibodies were detected by incubation with 1:500 dilutions of biotin-conjugated rat monoclonal anti-mouse IgG, IgG1, and IgA antibodies (BD Pharmingen^TM^, San Diego, CA, USA) at RT for 5 h followed by 2.5 mg/ml avidin-peroxidase (Sigma-Aldrich) for 30 min. The peroxidase substrate TMB (3,3′,5,5′-tetramethylbenzidine, Kierkegaard and Perry Laboratories, Gaithersburg, MD, USA) was added to each well and the optical density was measured by a Multiskan Spectrum ELISA reader (Thermo Scientific, Vantaa, Finland) at 450 nm. ELISA was performed for each isotype using the same protocol.

### Proliferation assay and cytokine analysis

At the end of the immunisation schedule, the mice were sacrificed and their spleens were harvested under sterile conditions and disrupted using a syringe. The erythrocytes were lysed, and the splenocytes were then suspended in complete medium [RPMI-1640 containing 10% foetal bovine serum, 25 mM HEPES, 2 mM L-glutamine, 100 U/ml penicillin, 100 µg/ml streptomycin, 1 mM sodium pyruvate, 5.5 × 10^−5^ M 2-mercaptoethanol and 0.1 mM non-essential amino acids, all from Hyclone Laboratories (Logan, UT, USA)]. The final cell concentration for proliferation analysis was 1 × 10^6^ cells/ml and cells were settled in flat-bottom 96-well plates (Costar Corporation, Cambridge, MA, USA). For cytokine and phenotypic analysis, cells were suspended at 2 × 10^6^ cell/ml in 5 ml tubes (Becton Dickinson, Franklin Lakes, NJ, USA). Cell cultures were stimulated with 5 μg/ml of CCE, 1 μg/ml of SA35 or 2.5 μg/ml of SA40 and incubated in 5% CO_2_ at 37 °C for five days. Cell cultures for negative controls were grown without any stimulants, whereas positive control cells were stimulated with 1.0 μg/ml of concanavalin A. Lymphocyte proliferation was measured after 12 h of culture by ^3^H-thymidine incorporation in the presence of 0.5 µCi/well [^3^H]-thymidine (Amersham Life Science, Buckinghamshire, UK) sampled in triplicate. Proliferation was expressed as a stimulation index (SI) (i.e. counts per minute of CCE-stimulated cells divided by counts per minute of unstimulated cells). An SI above 3.0 was considered positive.

For the cytokine analysis, culture supernatants were harvested after five days of culture and stored at −70 °C until assayed. Cytokine levels (IFN-γ, IL-4, IL12) were measured in culture supernatants by ELISA. Essentially, 96-well plates (Nunc-Immuno^TM^ Plate MaxiSorp^TM^ Surface, Sigma-Aldrich, Roskilde, Denmark) were coated with a solution of 1 μg/ml (in 0.1 M Na_2_HPO_4_, pH 9.0) of monoclonal rat anti-mouse cytokine antibody (BD Pharmingen). After overnight incubation at 4 °C, the plates were washed with PBS-T and blocked with 1% BSA (Sigma-Aldrich) in PBS for 2 h at 37 °C. After washing, serial dilutions of culture supernatants and standards, i.e. recombinant mouse cytokines, were added to the wells and incubated overnight at 4 °C. The plates were then washed and the appropriate biotin-conjugated rat anti-mouse cytokine antibody (BD Pharmingen) was added to the well and incubated for 2 h at 37 °C. After washing and the addition of HRP-conjugated streptavidin (BD Pharmingen) for 2 h at 37 °C, the TMB (Kirkegaard & Perry Laboratories) substrate was added to the wells and absorbance was measured at 450 nm after 10–20 min. Standard curves were generated for each cytokine using the corresponding murine recombinant standard. The minimum detection level for each cytokine by ELISA was < 15 pg/ml for IFN-γ, < 4 pg/ml for IL-4, and < 15 pg/ml for IL-12.

### Lymphocyte phenotypic analysis

To analyse the lymphocyte subsets after incubation with CCE, the splenocyte suspension from each mouse was washed with PBS containing 2% BSA, and further re-suspended in 100 µl of the same buffer. The cells were then incubated with 5 µl of the following fluorescein isothiocyanate (FITC) or phycoerythrin (PE) conjugated monoclonal antibodies to murine leukocyte differentiation molecules: anti-CD3, anti-CD4, anti-CD8, anti-CD25, and CD30 (BD Pharmingen). The cells were analysed for single and dual fluorescence assay in a fluorescence-activated cell-sorter (FACScalibur; Becton Dickinson, Franklin Lakes, NJ, USA) and data were acquired by CELL-Quest software (Becton Dickinson). The instrument was set to measure the fluorescence intensities of forward-angle light scatter (FSC), side-angle light scatter (SSC-H), FITC (FL1), PE (FL2) and FITC (FL1). Cells incubated with FITC- and PE-conjugated mouse IgG1/IgG2a served as isotype control. The proportion (%) of cells expressing a given molecule was determined as the average of three replicas.

### Mucosal immunisation of female mice

**Fig. 1:**
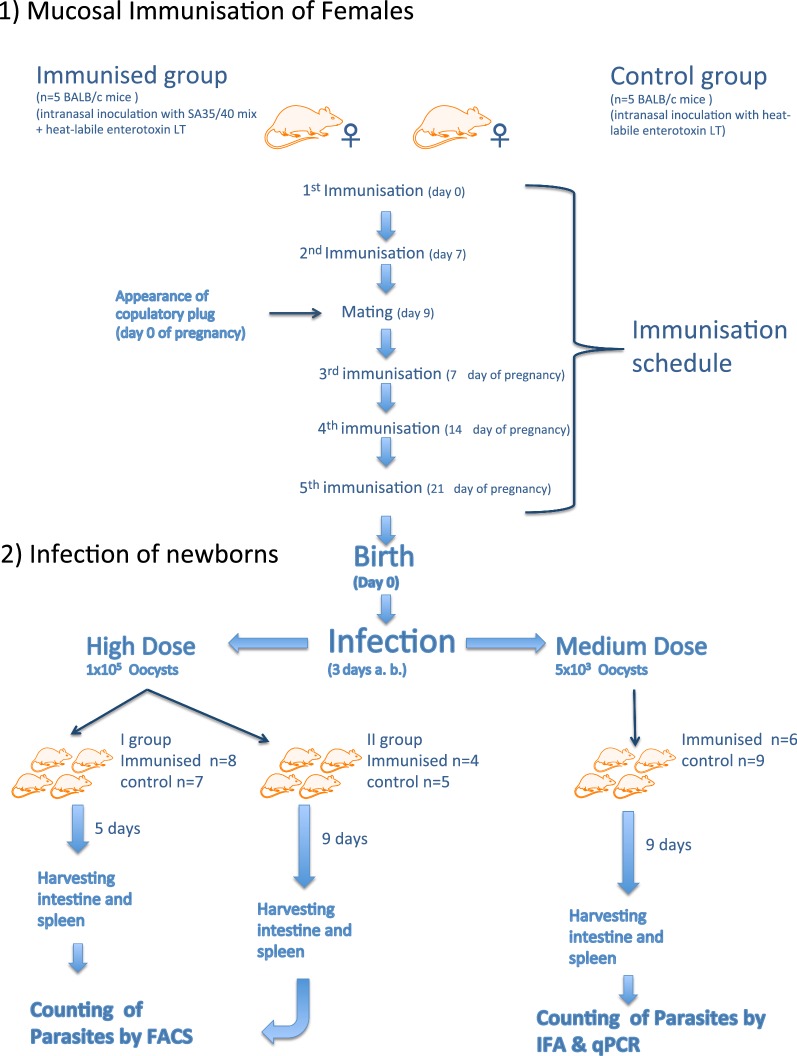
Schematic drawing of the maternal immunisation schedule and the infection model applied in this study

Two groups (experimental and control groups) of five mice each were anaesthetised with ketavet (100 mg/ml) and xylazine (20 mg/ml) at 50 mg/kg and 3 mg/kg, respectively. The mice from the experimental group were then immunised intranasally with 15–20 μl of PBS containing 1 μg of LT with 5 μg of SA35/40 mix, whereas the mice from the control group were inoculated intranasally with 15–20 μl of PBS containing 1 μg of LT. After seven days, the same immunisation protocol was repeated. Then, bedding from cages housing male mice was transferred to the cages housing females for 48 h to induce oestrus. Subsequently, one male and two females were kept in the same cage for 72 h and the females were examined daily for the presence of copulatory plugs. The day of plug detection was assumed to be day 0 of pregnancy [27]. Immunisations were repeated at days 7, 14, and 21 of pregnancy (Fig. 1). Blood samples were taken by tail bleeding on days 0, 7, 14, 21, 28, 35 and 230 after initial immunisation. Individual sera were stored at −20 °C until analysis. Faecal samples were collected at the same times and treated and stored as described above. Specific IgG and IgA to *C. parvum* were measured in serum and faecal samples by ELISA. Proliferation assays and phenotypic analysis of spleen cells were performed as described above.

### Experimental infection of newborn mice

To study the protection induced by mucosal immunisation, six litters of three-day-old BALB/c mice (39 mice) were used. Two litters, which were born from mothers immunised intranasally with SA35/40 with LT, were infected with 10^5^ oocysts and then sacrificed five [28] and nine days post-infection (p.i.), respectively [29]. Another litter born from an intranasally immunised mother was infected with 5 × 10^3^ oocysts and sacrificed at nine days p.i. The remaining three litters were the respective control groups. All suckling mice were experimentally infected by the oral route using oocysts suspended in 20 µl of PBS with a 24-gauge gavage needle and kept with their mothers. Suckling mice were anaesthetised before euthanasia and the whole intestine was collected. Lymphocytic phenotypic analysis was performed on spleens from mice born from intranasally immunised mothers, infected with 10^5^ oocysts and sacrificed five days later.

### Quantification of oocysts and intracellular forms of *C. parvum* in the intestinal content and ileum of newborn mice

The entire intestinal content was collected after washing of the intestinal tract with PBS. Samples were vortexed for 30 s with a 1-min pause. The supernatant was centrifuged at 2500×*g* for 10 min at 4 °C. The pellet containing oocysts and free forms of the parasite was then suspended in 1 ml of PBS and vortexed for 30 s. Each sample was divided in two aliquots: one aliquot was tested by an immunofluorescence assay, i.e. flow cytometry or indirect immunofluorescence assay (IFA), whereas the second aliquot was used to extract DNA for qPCR (see below).

Using flow cytometry, the number of oocysts in the faecal sample was evaluated using an acquisition gate based on the forward-angle light scatter (FSC-H) characteristic and the fluorescence intensities of FITC (on FL1 detector) of a positive control [30, 31]. The positive control consisted of an oocyst suspension in PBS incubated with the anti-*C. parvum* IgG. Flow cytometry analysis was performed three times for each mouse and the number of oocysts was expressed as the mean ± SD for each mouse group. Intracellular parasites were counted in the ileum homogenate from each mouse [28, 32]. Homogenisation was carried out individually in 1 ml of PBS for 1 min at 20×*g* using a potter homogeniser and further diluted 1:5 in PBS. The suspension was then filtered using a 30-µm Filcon syringe (Beckton Dickinson) and incubated with the anti-*C. parvum* IgG and an anti-rabbit FITC antibody for 30 min at RT. Intracellular parasites were counted using flow cytometry as above. The number of intracellular stages was expressed as the mean ± SD for each mouse group. When significant differences between the experimental and control groups were not found, the number of oocysts was also evaluated using IFA (see below).

For IFA, the oocyst suspension was mixed with 20 µl of a solution of 0.5 µg/ml anti-*C. parvum* IgG in PBS. The mixture was kept in the dark at 4 °C for 30 min and then centrifuged for ten min at 2500×*g*. The pellet was resuspended in 1 ml of PBS, and 20 µl of an anti-rabbit FITC antibody was added. The samples were kept in the dark at 4 °C for 30 min and then centrifuged at 2500×*g* for 10 min. The pellet was suspended in 1 ml of PBS and transferred to a polystyrene tube and kept at 4 °C. All samples were analysed on the day of oocyst collection. The number of oocysts was evaluated by fluorescence microscopy (Zeiss Axioplan 2; Carl Zeiss, Jena, Germany) at 400× magnification [31].

### Histological examination of the ileum of newborn mice

After washing (see above), the intestines from mice infected with 5 × 10^3^ oocysts were fixed in 4% formaldehyde in PBS for 4 h for histological examination, which was performed using the following procedure. The ileum portion was cut and washed in tap water overnight, dehydrated in increasing concentrations of ethanol from 50 to 100%, placed in 100% xylol and finally included in paraffin. The paraffinated tissue was cut using a Leica RM2125 RTS microtome (Leica Biosystems, Nussloch, Germany) into microscopic slices of 4–6 μm, distributed on microscopic slides and dried overnight at 37 °C. The slices were then deparaffinated by immersion in 100% xylol for 5 min and re-hydrated with decreasing concentrations of ethanol from 100 to 50% and a final wash in distilled water for 2 min. Antigen retrieval from fixed tissue was performed using the following “pressure cooking protocol”: the microscopic slides were placed in a stainless steel slide rack in the boiling citrate buffer (10 mM sodium citrate, 0.05% Tween 20, pH 6.0) in a pressure cooker heated by a hot plate; the lid was then closed for steaming at high pressure for 90 s, and the pan was then rapidly cooled using running tap water on the lid. Immediately after the slides were prepared for indirect immunofluorescence assay (IFA), blocked using 2% foetal calf serum (FCS) in PBS for 1 h at room temperature, incubated for 1 h with anti-*C. parvum* rabbit serum [26] diluted 1:500 in 2% FCS in PBS, and washed three times (5-min wash) with PBS to remove unbound antibodies. Secondary incubation was conducted for 1 h at room temperature using goat anti-rabbit antibody conjugate with fluorescein (FITC) (BioRad, Hercules, CA, USA) diluted 1:1000 in 2% FCS in PBS, and the slides were then washed as above and sealed using ProLong Antifade (Thermo Fisher Scientific, Waltham, MA, USA). Microscopic inspection of the tissue slices was conducted using a fluorescence microscope (Zeiss Axioplan 2) at 1000× magnification and digital images were obtained using AxioVision software (Carl Zeiss).

### Quantitative PCR (qPCR) of intestinal content

For qPCR, aliquots of whole intestinal content from mice infected with 5 × 10^3^ oocysts (see above) were centrifuged, suspended in 1 ml of 50% ethanol in PBS and stored at −20 °C until DNA extraction. Each aliquot was centrifuged, washed once with PBS and resuspended in 100 µl of lysis buffer (10 mM DTT, 1.8 mg/ml proteinase K in PBS), transferred to a 96-well plate and incubated for 1 h at 55 °C. For automated extraction, the plates were transferred to a Biosprint 96 apparatus (Qiagen, Hilden, Germany) using the protocol for blood samples and One-For-All Vet Kit reagents (Qiagen), and DNA was eluted in a final volume of 50 µl. DNA was also extracted in the same way from referenced dilutions of purified oocysts in triplicate to obtain samples with 10^5^, 10^4^, 10^3^, 100, 10, 1 and a theoretical 0.1 oocysts, and used for a standard curve based on the median crossing threshold (CT) in qPCR experiments. DNA samples from infected mice and referenced dilutions were analysed using real-time PCR assay (qPCR) based on the COWP gene [33]. This assay was tested for sensitivity using reference samples, obtaining positive curves with a single oocyst (CT = 36 in three out of three replicas) and up to a theoretical 0.1 oocysts (CT = 39 in one out of three replicas), and samples producing less than 40 CT were considered positive. COWP primers were synthesised by Primm (Milan, Italy), and TaqMan probe and COWP probe labelled with 5′-hexachlorofluorescein (HEX; λ_em_= 553 nm) were from TIB Molbiol (Berlin, Germany). Each PCR reaction was completed in a final volume of 25 μl, comprising 12.5 μl of LightCycler 480 Probes Master mix containing FastStart Taq DNA polymerase, a proprietary buffer including deoxynucleotide triphosphates (dNTPs), MgCl_2_ at a final concentration of 3.2 mM, 300 nM of each primer, 500 nM of TaqMan probe, and 5 µl (1/20 of whole intestinal content) as DNA sample.

All reactions were performed on a 96-well plate in a LightCycler 480 PCR system (Roche Diagnostics, Mannheim, Germany) in triplicate and three negative controls were included in each plate. The PCR conditions were: 10 min of incubation at 95 °C followed by 40 cycles of 95 °C for 15 s and 60 °C for 1 min. Fluorescence data (three data real-time points) were collected at the end of each cycle.

### Statistical analysis

To compare each experimental group with the relevant control group, the Mann-Whitney U-test was used. A *P-*value < 0.05 was considered significant.

## Results

### Intraperitoneal (IP) immunisation of adult BALB/c mice with SA35 and SA40 peptides and analysis of immune response

**Fig. 2:**
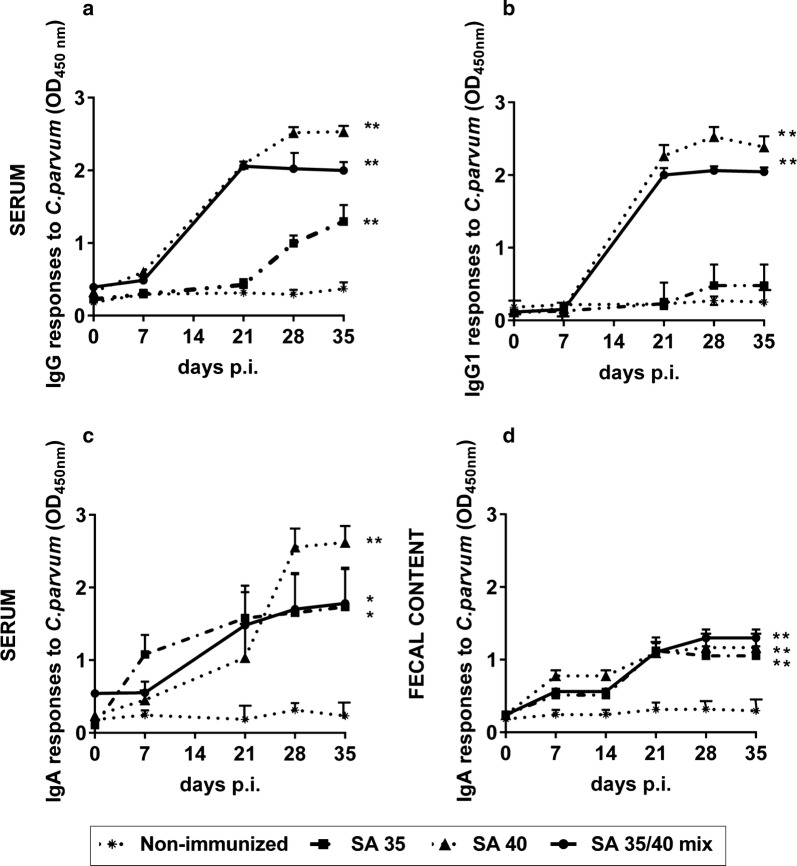
Kinetics of the IgG (**a**), IgG1 (**b**) and IgA (**c**) responses to *Cryptosporidium parvum*-crude extract (CCE) in BALB/c mouse sera. **d** Kinetics of the IgA responses to CCE in BALB/c mouse faecal content. Square dots: mice intraperitoneally (IP) immunised with SA35 peptide; triangular dots: mice IP immunised with SA40 peptide; circular dots: mice IP immunised with SA35/40 mix; asterisks: mice (naive) IP inoculated with phosphate buffered saline (PBS). Dots represent mean values of five mouse serum samples in **a**–**c** and mean values of five mouse faecal content samples in **d**. Bars represent standard error (SE). Significance values (*P < 0.05; **P < 0.01) were calculated between experimental and control groups at 35 days PI

The IP immunisation of adult BALB/c mice to a single antigen (SA35 or SA40) or to a mixture of the two antigens (SA35/40 mix) induced specific anti-*Cryptosporidium* IgG in serum after day 14 following initial administration. Later, the level of specific IgG progressively increased over time, reaching the highest response 35 days after initial immunisation (SA35, *U*_(3)_ = 4, *Z* = −1.88004, *P* = 0.03; SA40, *U*_(0)_ = 4, *Z* = −2.550672, *P* = 0.006; SA35/40 mix, *U*_(0)_ = 4, *Z* = −2.50672, *P* = 0.0064; Fig. 2a). SA40 alone and the SA35/40 mix induced mainly IgG1 (SA40 and SA40, *U*_(0)_ = 4, *Z* = −2.50672, *P* = 0.0064; Fig. 2b). Specific anti-*Cryptosporidium* IgA were detected in sera from day 7 after initial immunisation, and then slightly increased until day 35 p.i. (*U*_(0)_ = 2, *Z* = −2.50672; for SA35 and SA35/40 mix, *P* = 0.0079, for SA40, *P* = 0.0159; Fig. 2c). As expected, sera from mice immunised with SA35, SA40 or SA35/40 mix reacted with their respective homologous antigens by ELISA (data not shown). Specific anti-*Cryptosporidium* IgA were detected in faeces from day 7 after initial immunisation until the last sampling (day 35) with all the three antigen formulations (*U*_(0)_ = 2, *Z* = −2.50672, *P* = 0.0079 for SA35, SA40 and SA35/40 mix; Fig. 2d). No specific IgA response was detected in faeces at day 0 in the control mice.

**Fig. 3:**
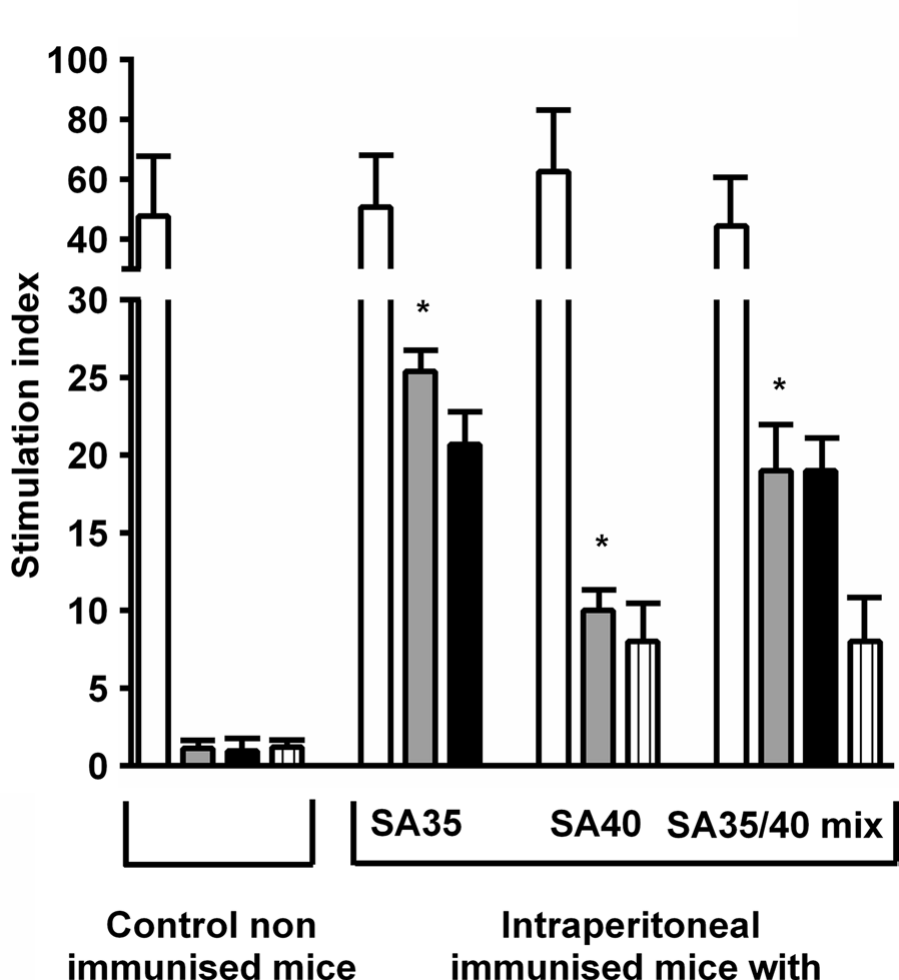
Splenocyte proliferation assay of non-immunised BALB/c mice (control group) and BALB/c mice intraperitoneally immunised with SA35 peptide, SA40 peptide or SA35/40 mix. Bars represent the mean of the stimulation index of splenocytes from five mice, small bars represent SE. Data are representative of three experiments. Splenocytes were stimulated *in vitro* with concanavalin A (white bars), *Cryptosporidium parvum*-crude extract (grey bars), SA35 peptide (black bars), or SA40 peptide (striped bars)

CCE induced proliferation in splenocytes from SA35, SA40 and SA35/40 mix in IP immunised mice. The highest responses to CCE were obtained in splenocytes from mice immunised with SA35 alone or the SA35/40 mix. Splenocytes from immunised and control mice responded to concanavalin A. Single peptides also induced proliferation in splenocytes from mice immunised with homologous peptides (Fig. 3). CCE induced IFN-γ production in the splenocytes from all groups of immunised mice. Splenocytes from mice immunised with SA35 or SA40 produced higher levels of IFN-γ after stimulation with homologous peptides than mice immunised with the SA35/40 mix and stimulated with the same mix. IL-12 production was observed in all mouse groups; the highest level of IL-12 production was observed in splenocytes from mice immunised with SA35. Minimal basal production of IL-4 was detected in spleen cells from some groups of immunised mice (Table 1).

**Table 1:** Interferon (IFN)-γ, interleukin (IL)-4 and IL-12-40p, produced by splenocytes from immunised mice

Immunised group	Stimulant	IFN γ		IL-4		IL-12 40p	
		Mean, pg/ml (range)	No. of mice^a^	Mean, pg/ml (range)	No. of mice^a^	Mean, pg/ml (range)	No. of mice^a^
SA 35	None	< 15 (< 15)	0/5	11 (< 4–27.13)	2/5	197 (24–436)	5/5
	CCE	81 (15–350)	2/5	23 (< 4–93.5)	2/5	274 (14–738)	4/5
	SA 35	1082 (15–1623)	5/5	59.3 (< 4–162)	3/5	522 (67–960)	5/5
SA 40	None	< 15 (< 15)	0/4	10 (< 4–16)	2/4	84 (19–260)	4/4
	CCE	45 (15–58)	4/4	7 (< 4–17)	1/4	44 (13–93)	3/4
	SA 40	692 (15–1605)	4/4	20 (< 4–52)	2/4	84 (18–202)	4/4
SA35-40 mix	None	< 15 (< 15)	0/5	20 (< 4–20)	1/5	25 (18–45)	5/5
	CCE	30 (15–80)	4/5	88 (< 4–88)	1/5	12 (4–18)	4/5
	SA 35/40 mix	26 (15–86)	5/5	34 (< 4–79)	3/5	62 (12–140)	4/5

*Notes*: Mice were immunised with SA35, SA40 peptides or SA35-40 mix. Splenocytes were cultured in absence of stimulating factor (None), or stimulated with *C. parvum* crude extract (CCE) or the same antigen preparation used for the immunisation (i.e. SA35 or SA40 or SA35/40 mix). Cytokines were assayed on the culture supernatant. Concentrations ≥ 15 pg/ml for IFN-γ, ≥ 4 pg/ml for IL-4 and ≥ 14 pg/ml for > IL-12 40p were considered positives

^a^No. positive/no. examined

**Fig. 4:**
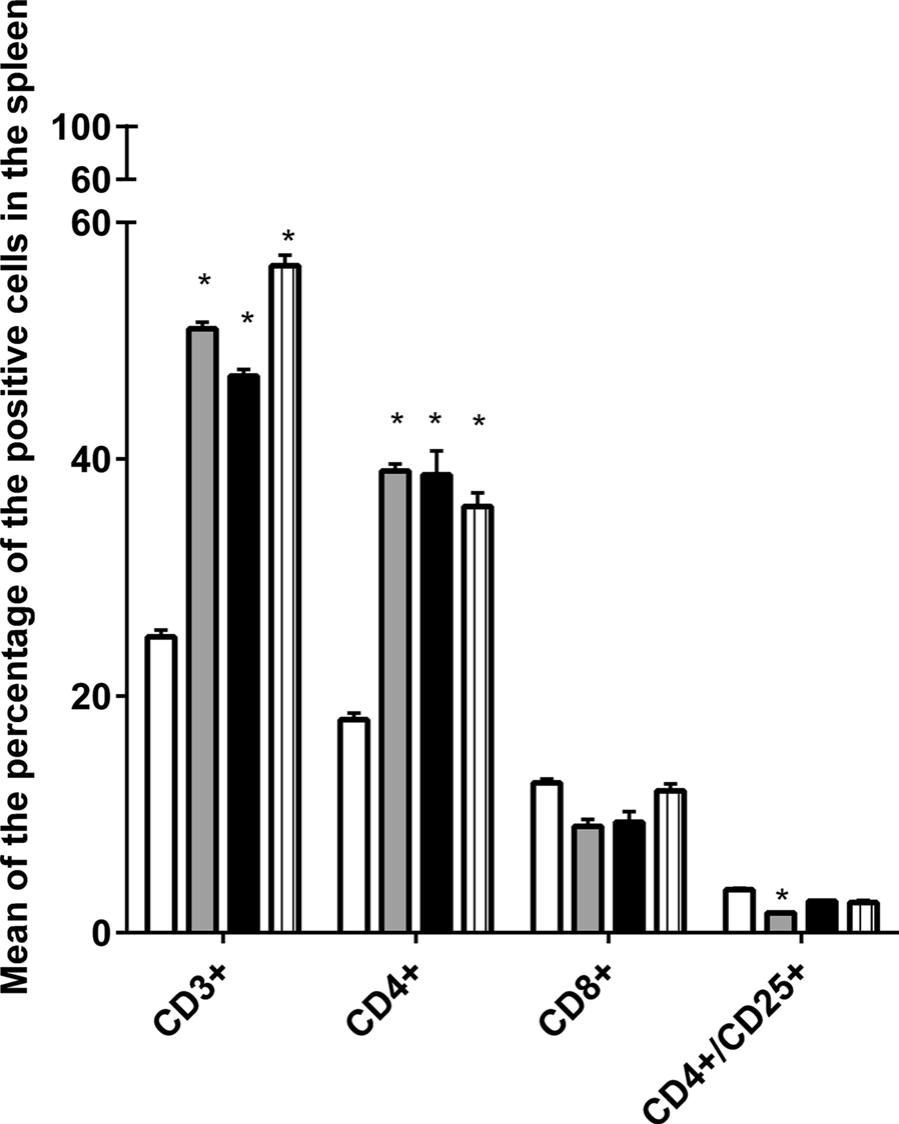
Percentage of positive cells in the spleen of non-immunised BALB/c mice control group (white bars) and BALB/c mice intraperitoneally immunised with SA35 peptide (grey bars), SA40 peptide (black bars) or SA35/40 mix (striped bars). Bars represent the mean of the percentages from five mice, small bars represent SE. Data are representative of three experiments. Significance values (*P < 0.05) were calculated between experimental and control groups

Induced cell populations in splenocytes were analysed at day 35 after initial immunisation. The percentages of CD3+ and CD4+ T cells were similar in mice immunised with SA35, SA40 and the SA35/40 mix and were significantly higher than in non-immunised control mice. However, the percentage of CD8+ T cells was similar among all groups, including the controls. Moreover, the percentage of activated CD4+ lymphocytes co-expressing the high affinity interleukin-2 receptor (CD25) was decreased in all immunised groups, particularly in the SA35 immunised group (*U*_(0)_ = 5, *Z* =, 2.80224, *P* = 0.0022; Fig. 4).

### Mucosal immunisation of female BALB/c with SA35/40 mix and experimental infection of their litters

**Fig. 5:**
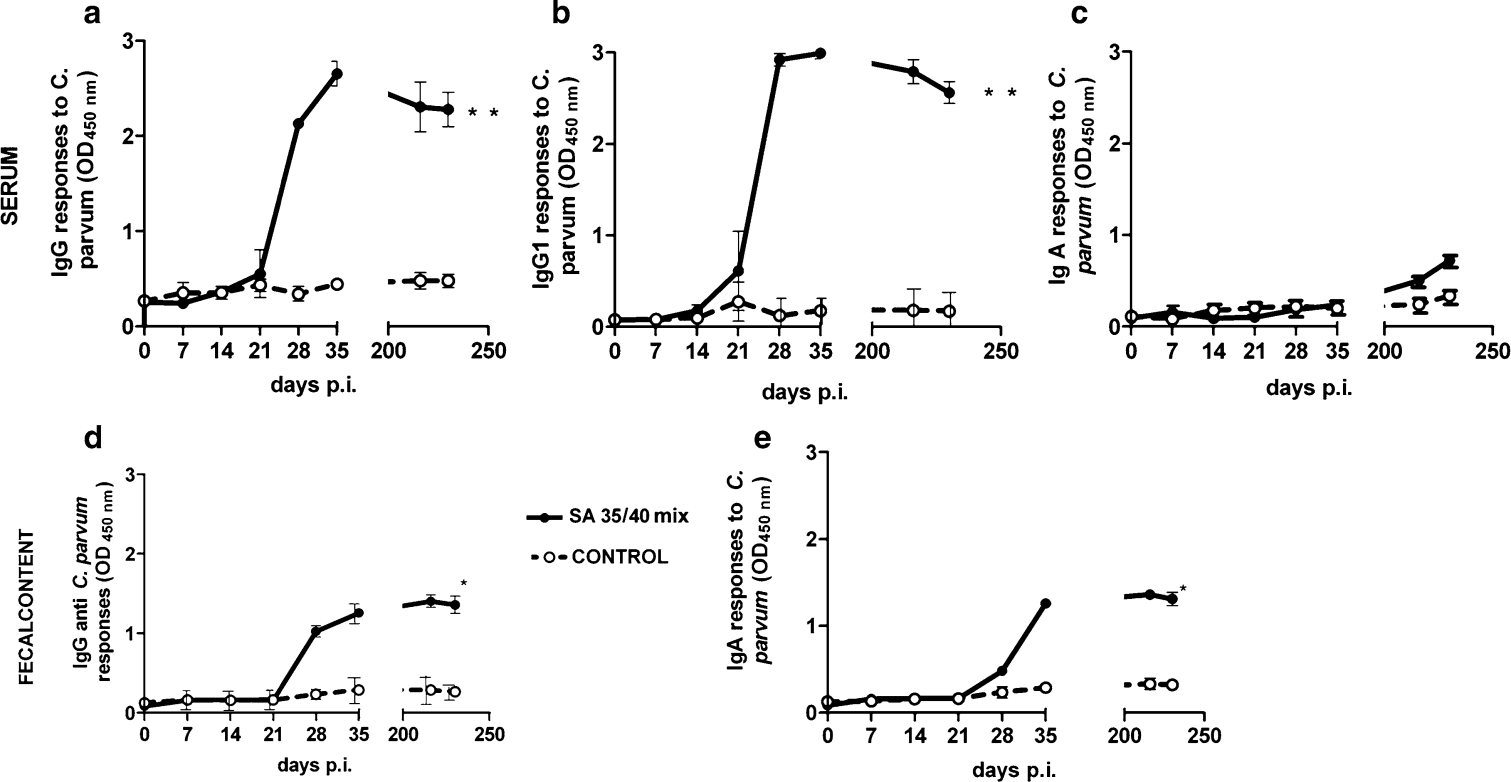
Kinetics of the IgG (**a**), IgG1 (**b**) and IgA (**c**) responses to *Cryptosporidium parvum*-crude extract (CCE) in BALB/c mouse sera. Kinetics of the IgG (**d**) and IgA (**e**) responses to CCE in BALB/c mouse faecal content. Black circles: mice were immunised by mucosal delivery to SA35/40 mix in the presence of LH. Open circles: mice were delivered intranasal phosphate buffered saline (PBS) and LH. Circles represent mean values of five mouse serum samples, small bars represent SE. Each determination was conducted in triplicate. Significance values (*P < 0.05; **P < 0.01) were calculated between experimental and control groups

The mucosal delivery of SA35/40 mix in female BALB/c mice induced specific anti-*Cryptosporidium* IgG (mainly IgG1) in serum 21 days after initial immunisation. The IgG level increased over time from day 28 until day 35. High IgG levels were detected until the last observation on day 230 (*versus* control group *U*_(0)_ = 2, *Z* = −2.50672, *P* = 0.01208 for IgG and IgG1; Fig. 5a, b). A low specific IgA level was detectable in serum until the end of the experiment (230 days after initial immunisation; Fig. 5c). In faeces, specific anti-*Cryptosporidium* IgG and IgA began to increase on the day 28 following the first delivery and then over time (*versus* control group *U*_(0)_ = 3, *Z* = 2.64733, *P* = 0.0022 and *U*_(0)_ = 5, *Z* = −2.80224, *P* = 0.00512 for IgG and IgA, respectively). No specific IgG or IgA responses were detected in faeces at day 0 or in the control mice (Fig. 5d, e).

**Fig. 6:**
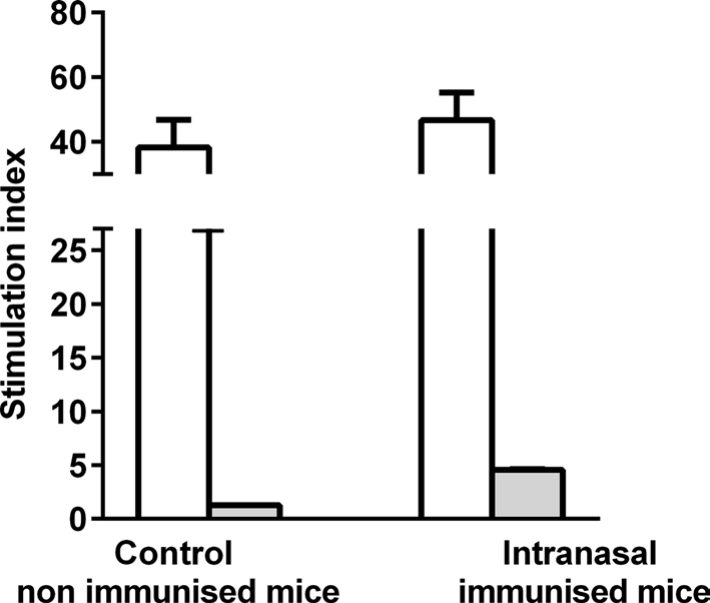
Splenocyte proliferation assay on non-immunised BALB/c mice (control group) and BALB/c mice intranasally immunised with SA35/40 mix. Splenocytes were stimulated *in vitro* with concanavalin A (white bars) and *Cryptosporidium parvum*-crude extract (grey bars). Bars represent the mean of the stimulation index of splenocytes from five mice, small bars represent SE. Data are representative of three experiments

CCE and ConA induced proliferation in splenocytes from mice immunised intranasally with the SA35/40 mix. Splenocytes from control mice responded only to concanavalin A (Fig. 6). The cytometric analysis was performed in CCE-stimulated splenocytes from both adult mice immunised intranasally and their litters. Spleens were harvested from adult mice at day 236 following initial immunisation. The percentages of CD3+/CD4+ and CD30+ T cells were increased in adult mice treated with the SA35/40 mix. The same results were obtained with the CCE-stimulated splenocytes from passively immunised litters infected with 10^5^ oocysts (Table 2).

**Table 2:** Phenotypic analysis in splenocytes from immunised mothers and their infected litters

Group	CD3^+^-CD4^+^ (%)	CD30+ (%)
BALB/c adults immunised with PBS + LH	27	1.8
BALB/c adults immunised with SA35/40 mix + LH	48	50
BALB/c mice born from mice immunised with PBS + LH	38.8	16
BALB/c mice born from mice immunised with SA35/40 mix + LH	61	44

*Notes*: Adult female BALB/c mice were mucosally immunised with SA35/40 mix. Cells were stimulated with *C. parvum* crude extract and were gated for 90–95% CD45^+^. Analysis of splenocytes from adult mice was carried out on the last day of the experiments. The number of mice is shown in Fig. 1. Newborn mice born from intranasally immunised mothers were infected with 10^5^ oocysts and sacrificed five days later

**Fig. 7:**
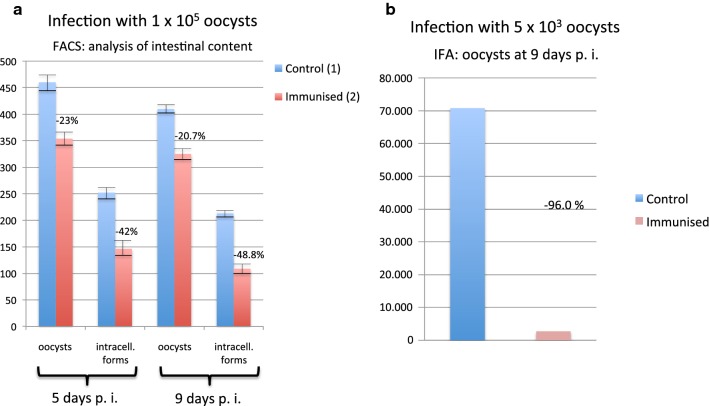
**a** Effect of maternal administration of SA35/40 mix on *C. parvum* infection on parasitic load of neonate mice. Infection with 1 × 10^5^ oocysts; bar graphs showing the number of parasites (× 1000) as oocysts in intestinal content and intracellular form in mice from not immunised mothers (control group) (light blue bars) and mice from immunised mothers (pink bars) at five days (left) and nine days PI determined using flow-cytometry. **b** Infection with 5 × 10^3^ oocysts; oocysts in intestinal content in control group (light blue bar) and mice from immunised mothers (pink bar) determined using immunofluorescence microscopy and manual oocyst counting

When infected with 10^5^ oocysts, passively immunised suckling mice showed similar levels of reduction at day 5 and day 9 following infection. In particular, a significant reduction of 23% of excreted oocysts (*U*_(20)_ = 38, *Z* = −2.76956, *P* = 0.0028) and 42% of intracellular forms (*U*_(14)_ = 23, *Z* = −2.43588, *P* = 0.00734) in the ileum was observed using flow cytometry (Fig. 7a).

**Fig. 8:**
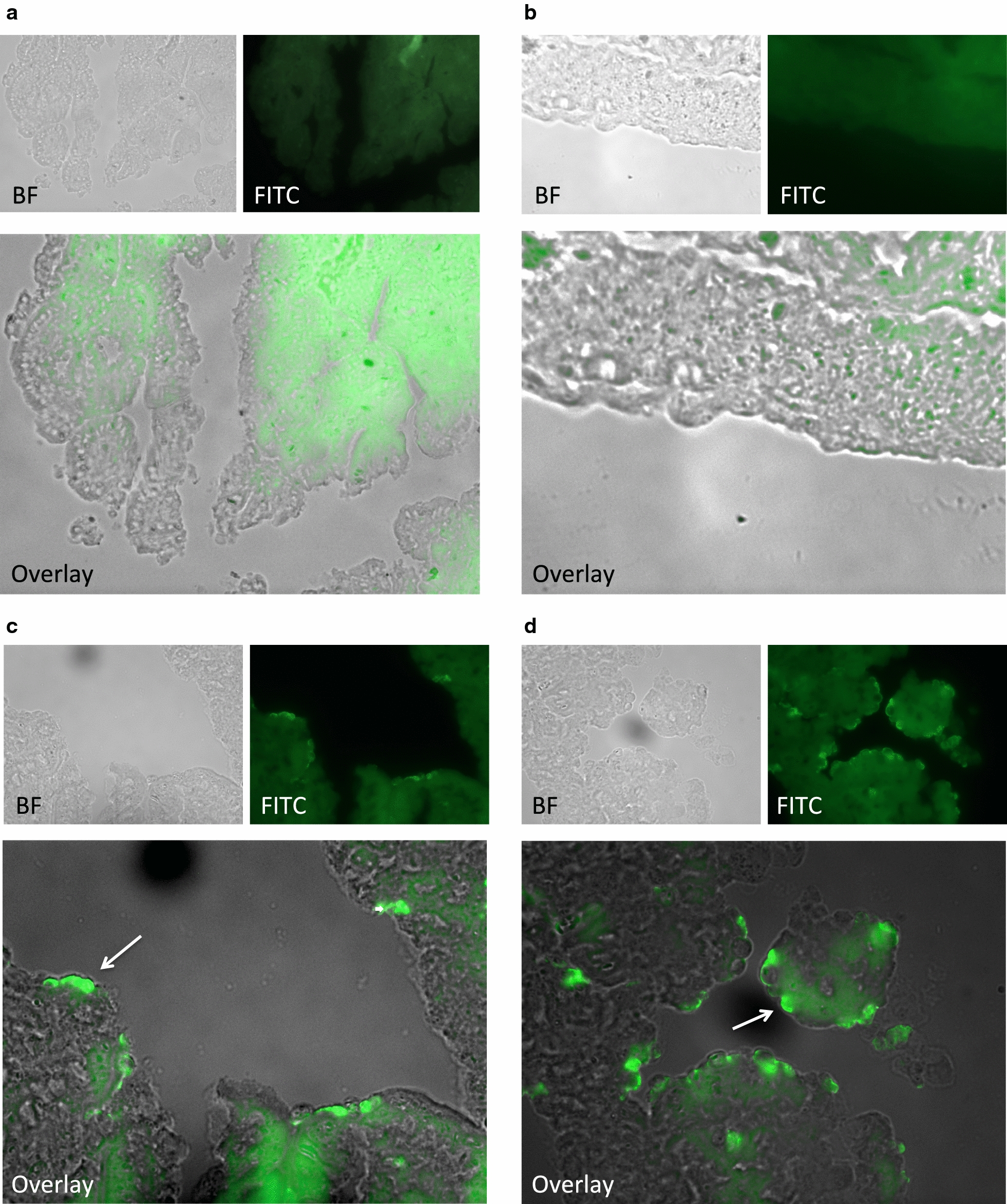
Effect of maternal administration of SA35/40 mix on *C. parvum* infection in the ileum of neonate mice. Transverse sections of small intestine from neonate mice after infection with 5 × 10^3^ oocysts labelled with anti-*C. parvum* rabbit serum [26] and a FITC-conjugated secondary antibody. **a**, **b** Sections from mice born from females immunised with SA35/40 mix; parasites are not visualised. **c**, **d** sections from mice born from females immunised only with LT as adjuvant (control group); various parasitophorous vacuoles are visualised by brilliant green staining. Magnification 1000×; microscopic slides were acquired using transmitted light bright field (BF), green fluorescence light (FICT) and images merged together (overlay). White arrows in panels **c** and **d** indicate typical parasitophorous vacuoles emerging from the intestinal epithelial tissue

Infection of neonates was also undertaken with a lower dose of oocysts in order to get closer to a natural infective dose using 5 × 10^3^ oocysts. The histological examination of the ileums of infected mice did not show any evidence of the infection (i.e. parasitophorous vacuoles) in the analysed sections from neonates born from immunised females (Fig. 8c, d). By contrast, newborns from non-immunised females had numerous parasitophorous vacuoles disseminated along the epithelial lining of the intestinal lumen (Fig. 8c, d). Indeed, a quantitative analysis of the entire intestinal content at day 9 p.i. showed a striking 96% reduction in parasites in newborns from immunised females (*U*_(4)_ = 12, *Z* = 2.65165, *P* = 0.00402), as evaluated by microscopic counting after IFA (Fig. 7b).

### Quantification of all *C. parvum* forms in the intestinal content of suckling mice using qPCR assay

To confirm the overall reduction in parasitic forms in passively immunised newborn mice infected with the lower infective dose (i.e. 5 × 10^3^ oocysts), the intestinal contents of these newborns were evaluated using a real-time qPCR assay.

**Fig. 9:**
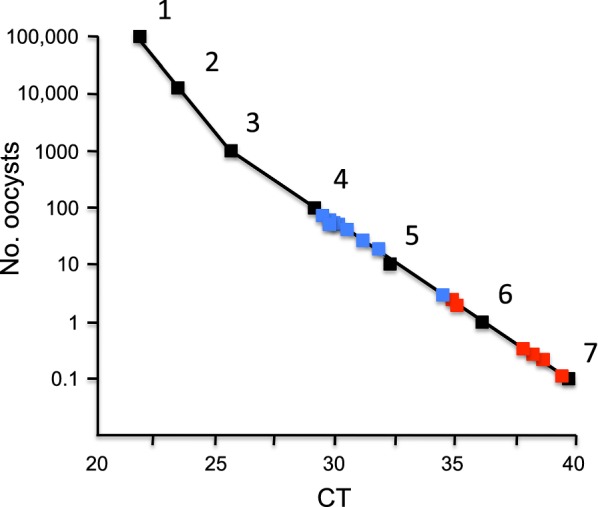
Quantification of COWP gene DNA copies by qPCR in the intestinal content of neonate mice infected with 5 × 10^3^ *Cryptosporidium parvum* oocysts. Values are expressed as the mean of cycles for crossing thresholds (CT), and each sample was tested in triplicate. Standard curve (black dots) was obtained from DNA extracted from the following oocysts dilutions: 1 × 10^5^ (1), 1 × 10^4^ (2), 1 × 10^3^ (3), 100 (4), 10 (5), 1 (6) and 0.1 (7). Blue dots represent CT values from control mice. Red dots represent CT values from immunised mice. Values from control mice are between 33 and 29 cycles, equivalent to DNA amounts from 6 to 100 oocysts, whereas values from immunised mice are between 39 to 35 cycles, equivalent to DNA amounts from 0.1 to 5 oocysts

As shown in Fig. 9, CT values in faecal samples from suckling mice born from immunised mothers ranged between 34 and 40, i.e. equivalent to 5–0.1 oocysts. Four out of 6 samples showed a CT value higher than 37, thus with less than one oocyst equivalent in their content. CT values for the control mice ranged between 27 and 33, i.e. equivalent to 10–100 oocysts. Therefore, qPCR analysis confirmed that the parasitic load in the intestinal contents of newborns from immunised mothers was extremely low when newborns were infected with the lower dose of 4.5 × 10^3^ oocysts.

## Discussion

Control of neonatal cryptosporidiosis is a challenging task because of the lack of appropriate drugs and the difficulties in providing effective immunisation in the early phase of life. Here, we explored the protective effect of anti-*C. parvum* maternal antibodies transferred to newborns through the placenta during gestation and through the colostrum in the first days of life. To this end, two recombinant peptides (SA35 and SA40) were first immunologically characterised by IP injection in adult mice to evaluate their potential as immunogens. Both peptides, separately and in a mixed formulation, induced specific IgG and IgA responses. SA35 and SA40 peptides have been previously described as immunodominant antigens involved in the maintenance of T-cell response in healthy *C. parvum*-sensitised individuals [20]. It is important to note that the amino acid sequences of SA35 and SA40 peptides do not show similarities with mammalian proteins, but, by contrast, are also highly conserved in other *Cryptosporidium* species and particularly in other human pathogens (data not shown). Therefore, it is most likely that antibodies for SA35 and SA40 also react with the strictly related proteins of *C. hominis* and *C. ubiquitum*. In this study, it has been demonstrated that IP immunisation with these recombinant peptides is able to support an effective cell-mediated immunity in response to specific stimuli: CCE-, SA35- and SA40-induced specific proliferation in splenocytes from all immunised adult mice. However, maximum proliferative responses to CCE were obtained in splenocytes from mice following IP immunisation with SA35 or the SA35/40 mix (Fig. 3). The induced cell populations showed a higher percentage of cells expressing CD3+ and CD4+. Moreover, splenocytes from mice immunised with SA35 showed a significant reduction in the percentage of regulatory cells (CD4+/CD25+) compared to the control group, whereas the others did not (Fig. 4). Overall, the three antigen formulations (SA35, SA40 or SA35/40 mix) induced IFN-γ, IL-12 and, to a lesser extent, IL-4 in immunised mice (Table 1). What is noteworthy is that stimulation with the homologous antigen resulted in the highest level of production of these cytokines even when the splenocytes also responded to stimulation with CCE (Table 1). Experimental studies and clinical cases have demonstrated that activation of CD4+ lymphocytes and production of IFN-γ are crucial for parasite clearance [34–37]. Indeed, IFN-γ production is one of the essential functions of CD4+ cells in response to *Cryptosporidium* antigens [38, 39]. With regard to humoral response, all three antigenic formulations (SA35, SA40 or SA35/40 mix) induced specific IgG, IgG1 and secretory IgA (Fig. 2). The production of these transferable antibodies is a fundamental requirement in conferring protective immunity for neonates. Therefore, the antigenic SA35/40 mix was used for mucosal immunisation of female mice in order to evaluate the protective effect on their offspring. The efficacy of this treatment was assessed on the basis of infection of littermates born from females immunised using two different doses of oocysts, 1 × 10^5^ and 5 × 10^3^. The comparison between *C. parvum*-infected littermates born from immunised mothers and their respective controls, i.e. *C. parvum*-infected littermates born from mothers immunised only using a mucosal adjuvant, showed that maternal protection is highly effective with the lower infective dose (5 × 10^3^), since there was a 96% reduction in parasitic burden (Fig. 7).

In adult females, mucosal immunisation with the SA35/40 mix leads to a long-lasting (33 weeks) humoral response at systemic and intestinal level (Fig. 5). Moreover, splenocytes from BALB/c mothers specifically proliferated in response to CCE and the resulting population showed an increase in the percentages of CD3+/CD4+ and CD30+ cells (Table 2).

The experimental infection of neonate mice born from immunised mothers showed that when neonates were infected with a massive dose of oocysts (1 × 10^5^), the protective effect on the progeny is measurable but ineffective (−23% of excreted oocysts). Conversely, a drastic reduction (96%) in oocyst emission was observed when neonates were infected with a lower dose (5 × 10^3^ oocysts), which still represents a huge amount given the small weight (about 1 g) of newborn mice (Fig. 7); in fact, this dose is comparable to a dose of 30 × 10^7^ oocysts in a 60 kg human. These oocyst numbers were tested in the first instance to allow a quantitative analysis of excreted oocysts and of all parasitic forms using flow cytometry, although the 5 × 10^3^ dose yielded parasite amounts below the sensitivity threshold for the instrument. To overcome this technical limitation, IFA/microscopic counting and qPCR were used to evaluate the minimal quantity of parasites in infected mice. Moreover, DNA samples for qPCR were prepared using an *ad-hoc* automated method in order to reduce handling errors.

Cell-mediated immunity showed unexpected signs of activity in splenocytes harvested from litters from immunised mothers, as there was an increase in the percentage of CD3+-CD4+ cells as well as CD30+ cells (Table 2). Similarly, T-cell responses were identified after maternal vaccination in newborn mice of a *Plasmodium yoelii* model, although there was no parallel increase in antibody response [40]. Recent studies indicate that during pregnancy, maternal molecules such as inflammatory cytokines, as well as microbial products, are transferred *in utero* to the foetus and this influences the foetal immune system [41]. Thus, neonatal hosts may compensate for an insufficiency in adaptive immune responses by having a heightened capacity to mount certain types of innate immune responses [42].

Attempts at passive immunisation through administration of hyperimmune colostrum have been performed in humans and in other mammals. Indeed, hyperimmune bovine colostrum was given to AIDS patients with prolonged cryptosporidiosis, resulting in a reduction in diarrhoea and an improvement in symptoms [15, 16]. In livestock, passive immunisation with recombinant antigens has shown a certain efficacy in protecting passively immunised calves and kids [43, 44]. More recently, heterologous protection was also successfully tested through administration of hyperimmune ovine colostrum in neonate mice [45]. Unlike previous studies, this study combined the use of a mixture of two defined antigens extensively characterised as immunogens. A further peculiarity of this study is the mucosal immunisation of females to confer protection for their progeny.

Maternal immunisation protocols for other pathogens have been applied successfully to prevent neonatal infectious diseases in various contexts. With regard to livestock, a vaccine for rotavirus, coronavirus and *Escherichia coli* (Rotavec-Corona^®^, MSD) is commonly used to immunise heifers and preparturient cows. This treatment induces a higher titre of specific antibodies for these pathogens in colostrum and milk [46] and has significant efficacy in reducing diarrhoea in neonate calves [47]. In humans, maternal immunisation with tetanus toxoid has been the first example of effective prevention for neonates [48] and it has been extensively applied as part of the WHO Maternal and Neonatal Tetanus Elimination (MNTE) program. Successful immunisations have been also achieved with influenza virus [49] and with the acellular pertussis antigen vaccine that protects infants from clinical pertussis [50]. Therefore, maternal immunisation can be an effective strategy to limit neonatal cryptosporidiosis before the development of a mature immune system.

## Conclusions

The SA35 and SA40 peptides enhance humoral and cell-mediated immune responses to *C. parvum* in adult mice. Mucosal immunisation of pregnant mice with a mixture of SA35 and SA40 antigens (SA35/40 mix) resulted in a remarkable reduction in parasite load in the gut of their infected offspring. Overall, this result represents the proof of concept that maternal immunisation can effectively prevent neonatal cryptosporidiosis.

## Data Availability

All data presented in this manuscript are available at the Istituto Superiore di Sanità.

## Abbreviations

WHO: World Health Organisation
US-FDA: US Food and Drug Administration
CpCDPK1: kinase inhibitors of *C. parvum* calcium-dependent protein kinase 1
CCE: *C. parvum*-crude extract
LT: heat-labile enterotoxin
IFA: immunofluorescence assay
MW: molecular weight
PBS: phosphate buffered saline
BSA: bovine serum albumin
RT: room temperature
TMB: 3,3′,5,5′-tetramethylbenzidine
SI: stimulation index
ELISA: enzyme-linked immunosorbent assay
IFN: interferon
Ig: immunoglobulin
IL: interleukin
FITC: fluorescein isothiocyanate
PE: phycoerythrin
FACS: fluorescence-activated cell-sorter
FSC: forward-angle light scatter
SSC: side-angle light scatter
qPCR: quantitative real-time PCR assay
CT: median crossing threshold
HEX: hexachlorofluorescein
TLR: toll-like receptor
